# Discrimination of Excited States of Acetylacetone through Theoretical Molecular-Frame Photoelectron Angular Distributions

**DOI:** 10.3390/molecules27061811

**Published:** 2022-03-10

**Authors:** Aurora Ponzi, Marin Sapunar, Nadja Došlić, Piero Decleva

**Affiliations:** 1 Department of Physical Chemistry, Ruđer Bošković Institute, 10000 Zagreb, Croatia; sapunarm@vscht.cz (M.S.); nadja.doslic@irb.hr (N.D.); 2 Istituto Officina dei Materiali IOM-CNR and Dipartimento di Scienze Chimiche e Farmaceutiche, Università degli Studi di Trieste, Via L. Giorgieri 1, 34127 Trieste, Italy

**Keywords:** photoelectron angular distribution, MFPADs, photoionization, photodynamics, excited states, acetylacetone

## Abstract

Photoelectron angular distribution (PAD) in the laboratory frame for randomly oriented molecules is typically described by a single anisotropy parameter, the so-called asymmetry parameter. However, especially from a theoretical perspective, it is more natural to consider molecular photoionization by using a molecular frame. The molecular frame PADs (MFPADs) may be used to extract information about the electronic structure of the system studied. In the last decade, significant experimental efforts have been directed to MFPAD measurements. MFPADs are highly characterizing signatures of the final ionic states. In particular, they are very sensitive to the nature of the final state, which is embodied in the corresponding Dyson orbital. In our previous work on acetylacetone, a prototype system for studying intra-molecular hydrogen bond interactions, we followed the dynamics of the excited states involved in the photoexcitation–deexcitation process of this molecule. It remains to be explored the possibility of discriminating between different excited states through the MFPAD profiles. The calculation of MFPADs to differentiate excited states can pave the way to the possibility of a clear discrimination for all the cases where the recognition of excited states is otherwise intricate.

## 1. Introduction

Photoemission observables of molecules in gas phase are generally measured in the laboratory frame (LF). Among these observables, photoelectron angular distributions (PADs) are particularly informative about the photoionization dynamics and the electronic character of the ejected photoelectrons [1,2,3].

Despite the informative character of PADs in the LF, the average over all molecular orientations that is required for their calculation leads to a loss of information on the partial-wave composition of the ionization continuum. Such information can be recovered by measuring PADs in the molecular frame (MF). In this case, the molecule has to be fixed with respect to the LF, commonly referred to as the alignment and orientation of molecules [4]. The resulting molecular-frame photoelectron angular distribution (MFPAD), which appears as highly structured and anisotropic, is much richer in information than the angular distribution obtained when randomly oriented molecules are considered.

Although the first theoretical suggestions to study PADs in the MF rather than in the LF appeared a long time ago [5,6,7], it was only in the last decade that burgeoning activity developed in the study of MFPADs, thanks to the considerable advancement of the light sources and detectors, as well as the theoretical modeling [8,9].

Due to this renewed interest, measurements and analysis of MFPADs contributed to an unprecedented degree of detailed information about molecular photoionization processes [10,11], electron–electron correlation [12], selection rules and localization of charge and core holes [13,14]. Among the applications of the fully differential photoelectron angular distribution measurements in the molecular frame, it was recently demonstrated that MFPADs are sensitive probes of the molecular bond length [15] and molecular structure [16].

In the case of core ionization, MFPADs also represent a suitable tool to examine the nature of the shape resonance [17,18,19], and to probe the presence of doubly excited states, imprinted in their profiles [20,21,22]. Moreover, they can be used to extract the photoelectron emission delay [23], avoiding the use of attosecond light pulses [24].

MFPAD measurements can be realized by orienting the target molecule at the instant of photoionization. In gas-phase studies, this can be done by means of a 3D laser alignment [25], by a mixed-field orientation approach [26] or by techniques, such as COLTRIMS, in which particles are measured in coincidence [27,28]. The coincidence method has been used mostly in the last decade in studies of one-photon ionization, taking advantage of the performance of third-generation synchrotron radiation facilities. Experiments on the MFPADs can be performed not only by using linearly polarized light but also with circularly or elliptically polarized light [29,30,31]. This permits the investigation of circular dichroism effects in the MF angular distributions and determining complete sets of matrix elements and phases.

More recently, new directions of research have emerged in pump-probe studies, where time-dependent photoionization of the target molecule often serves as a probe of dissociation or rearrangement processes launched by the pump pulse [32]. Recording the photoemission of the evolving molecular system in the MF allows one to remove the blurring due to the random orientation of the parent molecule, providing a comprehensive description of the studied chemical reaction driven by nonadiabatic couplings, and characterized by electron localization or charge migration [33].

Following our previous study on the photoexcitation–deexcitation of acetylacetone [34], we aim to investigate the possibility of discriminating between the different excited states on the basis of their MFPAD profiles. Let us recall that in the experiment of Squibb and co-workers [34], the enol form of acetylacetone was initially excited to the S${}_{2}\left(\pi \pi^{*}\right)$ state by a 266 nm (4.66 eV) pump pulse and the dynamics was monitored by a 64.4 nm (19.2 eV) ionizing probe pulse. While the lifetime of the S${}_{2}\left(\pi \pi^{*}\right)$ could be unambiguously determined from the experiment, this was not the case for the S${}_{1}\left(n\pi^{*}\right)$, T${}_{2}\left(\pi \pi^{*}\right)$ and T${}_{1}\left(n\pi^{*}\right)$ states, which are populated during the dynamics. The lifetimes of these states could not be extracted from the experiment simply because, in acetylacetone, the ionization energies for different processes (vide infra) are similar and give rise to the same experimental peak.

In the following, we present theoretical MFPADs associated with the singlet and triplet states involved in the relaxation mechanism of acetylacetone. Since MFPADs are highly characterizing signatures of the final states, we expect to infer information on the character of the excited species from the analysis of the corresponding electron angular distributions.

## 2. Materials and Methods

### 2.1. Cross-Sections in the Molecular Frame: MFPADs

Two reference systems have to be considered to treat the setup in which the molecule has a completely fixed orientation. The first is the laboratory frame, defined by the photon beam and the detection apparatus; the second is the molecular frame, which is fixed with respect to the molecule. The relative orientation of the two reference systems is determined by the Euler angles Ω=(α,β,γ). In order to directly obtain the MFPAD cross-sections for a fixed orientation Ω, one has to express the wavefunctions, the photon orientation and the electron momentum vector $\vec{k}$ in the MF.

By considering the photoemission process from a fixed-in-space molecule, the differential cross-section for the electronic ionic state *i*, in the commonly used dipole approximation, is given in atomic units by Equation (1):

(1) $$\frac{d^{2}\sigma_{I}\left(\omega \right)}{d\vec{k}d\Omega }=4\pi^{2}\alpha \omega |\langle \Psi_{I\vec{k}}^{-}|d_{1m_{r}}|\Phi_{i}{\rangle |}^{2}$$

where ω is the photon energy, $\vec{k}$ is the photoelectron momentum in MF, α is the fine structure constant, $\Psi_{I\vec{k}}^{-}$ is the final state of the system, characterized by the quantum numbers *I*, representing the remaining ion and a photoelectron with a well-defined momentum $\vec{k}$, *d*${}_{1m_{r}}$ refers to the field component in LF, and $\Phi_{i}$ is the initial state of the system.

We work in a single-channel approximation, where $\Psi_{I\vec{k}}=\hat{A}\Psi_{I}^{N-1}\phi_{\vec{k}}$, so that the dipole matrix elements reduce to the single particle expression $\langle \phi_{\vec{k}}|d_{1m_{r}}|\phi_{I}^{D}\rangle$, where $\phi_{I}^{D}$ is the Dyson orbital relative to the final ionic state $\Psi_{I}$. The Dyson orbital is defined as the overlap of the wavefunctions associated with the bound *N* and (*N*−1) systems. A thorough description of the Dyson orbital approach for the computation of photoionization observables has been reported in previous publications [35,36].

The dipole operator defined in the length gauge in LF is given by Equation (2):

(2) $$d_{1m_{r}}=\sqrt{\frac{4\pi }{3}}rY_{1m_{r}}$$

with $m_{r}=0$ for linear polarization and $m_{r}\pm 1$ for left or right circularly polarized light, respectively.

The dipole matrix elements in Equation (1) can be evaluated in the MF by expressing the dipole operator in LF through its MF components $d_{1\lambda }$ by the rotation (Equation (3)):

(3) $$d_{1m_{r}}=\sum_{\lambda }d_{1\lambda }^{MF}R_{\lambda m_{r}}^{1}\left(\Omega \right)$$

where $R_{\lambda m_{r}}^{1}\left(\Omega \right)$ is the Wigner rotational matrix.

After transforming the dipole operator in LF into the MF, one has to define the expressions for the initial and final states involved in Equation (1). The continuum wave function of the photoelectron with momentum $\vec{k}$, normalized to incoming wave S matrix conditions, can be expanded in partial waves as follows (Equation (4)):

(4) $$\phi_{\vec{k}}^{-}=\sum_{lm}i^{l}e^{-i\sigma_{l}}Y_{lm}^{*}(\vec{k})\phi_{Elm}^{-}$$

where $\sigma_{l}$ is the Coulomb phase shift and *E* corresponds to the electron kinetic energy, i.e., $E=\frac{k^{2}}{2}$, and the corresponding dipole transition moments (which are the ones actually computed) as (Equation (5)):

(5) $$D_{lmm_{r}}^{\left(-\right)}=\langle \phi_{Elm}^{-}|d_{1m_{r}}|\phi_{I}^{D}\rangle .$$

A well-known angular momentum development leads to the general result for the differential cross-section (Equation (6)) [2]:

(6) $$\frac{d^{2}\sigma_{I}\left(\omega \right)}{d\vec{k}d\Omega }=4\pi^{2}\alpha \omega {\left(-1\right)}^{m_{r}}\sum_{LM}A_{LM}Y_{LM}(\vec{k})$$

with $A_{LM}=A_{LM}\left(E,\Omega ,m_{r}\right)$, namely (Equation (7)):

(7) $$\begin{array}{cc} A_{LM} & =\sum_{lm\lambda ,l^{\prime }m^{\prime }\lambda^{\prime }}{\left(-1\right)}^{m+\lambda }\sqrt{\frac{\left(2l+1\right)\left(2l^{\prime }+1\right)\left(2L+1\right)}{4\pi }}\\ \; & \times \left(\begin{array}{ccc} l & l^{\prime } & L\\ -m & m^{\prime } & M \end{array}\right)\left(\begin{array}{ccc} l & l^{\prime } & L\\ 0 & 0 & 0 \end{array}\right)\\ \; & \times D_{lm\lambda }^{\left(-\right)}D_{l^{\prime }m^{\prime }\lambda^{\prime }}^{(-)*}\sum_{J}\left(2J+1\right)\left(\begin{array}{ccc} 1 & 1 & J\\ \gamma^{\prime } & -\gamma & \gamma -\gamma^{\prime } \end{array}\right)\\ \; & \times \left(\begin{array}{ccc} 1 & 1 & J\\ -m_{r} & m_{r} & 0 \end{array}\right)R_{\lambda -\lambda^{\prime },0}^{J}\left(\Omega \right) \end{array}$$

with the restriction due to 3*j* coefficients J=0,1,2.

The MFPADs can then be analyzed as polar plots of the differential cross-sections.

### 2.2. LCAO B-Spline Code

The linear combination of atomic orbitals (LCAO) B-spline code, based on the density functional theory (DFT) method, is used for the evaluation of eigenvectors in the continuum spectrum. A complete treatment of the method can be found in [37]. Here, we only report the main steps, consisting of: (i) a standard DFT calculation to obtain the ground-state electronic density [38,39]; (ii) construction of the Hamiltonian matrix in the LCAO basis set, followed by a generalized diagonalization for bound states and application of the Galerkin approach for continuum states [40]; (iii) dipole transition moment calculation to compute photoionization observables.

In the present method, both bound and continuum orbitals are expanded in a basis (Equation (8))

(8) $$\chi_{ilm}=\frac{1}{r}B_{i}\left(r\right)Y_{lm}\left(\theta ,\phi \right)$$

composed as a product of radial B-spline functions, $B_{i}\left(r\right)$, defined over a grid in the interval [0, R] and real spherical harmonics, $Y_{lm}\left(\theta ,\phi \right)$. The basis includes a one-center expansion, where the functions are centered on a single origin, together with a number of off-center functions, located at non-equivalent nuclei. This multicenter approach ensures the accurate treatment both of bound states and the continuum functions.

After building the Kohn–Sham Hamiltonian matrix with the ground-state density, bound states are then obtained by a standard diagonalization of the Hamiltonian matrix. The continuum solutions are calculated at fixed energies as the vectors associated with the lowest eigenvalues of the energy-dependent $A^{T}A$ matrix, where A(E)=(H−ES), with *S* being the overlap matrix [40]. In order to obtain normalized continuum orbitals, K-matrix boundary conditions are used, and dipole matrix elements are then transformed into incoming wave boundary conditions.

### 2.3. Computational Details

A standard DFT calculation has been performed with the Amsterdam Density Functional (ADF.2016) program [38,39] for obtaining the Self-Consistent Field (SCF) initial density to build the Hamiltonian matrix in the new basis. In this calculation, all atoms are described by a double zeta polarized basis (DZP) and the LB94 functional has been used to describe exchange and correlation effects [41]. The choice of this functional is well supported by a significant number of accurate photoionization studies performed over the years [42,43,44]. The parameters used to calculate bound and continuum orbitals expressed in the B-spline LCAO basis have been chosen to reach convergence with the lowest computational cost. To this end, we set the angular momentum to 20 for the long-range one-center expansion and we define a radial interval up to R${}_{max}$ = 25 a.u. with a step size of 0.2 a.u. To obtain bound states of sufficient accuracy, we added several off-center functions around the oxygen, carbon and hydrogen atoms, with L${}_{max}$ = 2 for O- and C-, and with L${}_{max}$ = 1 for H-atoms.

Both excited and ionic states have been obtained through Complete Active Space Self-Consistent Field (CASSCF) calculations with cc-pVDZ as the basis set, i.e., the same basis set used in the previous study [34]. In these calculations, we considered 22 molecular orbitals (MOs) in the irreducible representation *a* (C${}_{1}$ symmetry) as doubly occupied MOs in all the configuration state functions. This number also includes 7 frozen MOs, i.e., C-atom and O-atom core orbitals. The active space is then composed of 10 electrons (9 in the case of ionization energies) in 10 MOs, divided as follows: 5 occupied orbitals (23a–27a) and 5 virtual orbitals (28a–32a).

In the present correlated single-channel approach, the initial and final bound states that define the Dyson orbitals correspond to CASSCF wavefunctions. The Dyson orbitals are computed through a code set up in our laboratories, based on the direct evaluation of the overlap between the CASSCF wavefunctions separately optimized for the ion and for the neutral molecule [35]. All the calculations have been performed with Molpro2010.1 [45].

## 3. Results and Discussion

**Figure 1:**
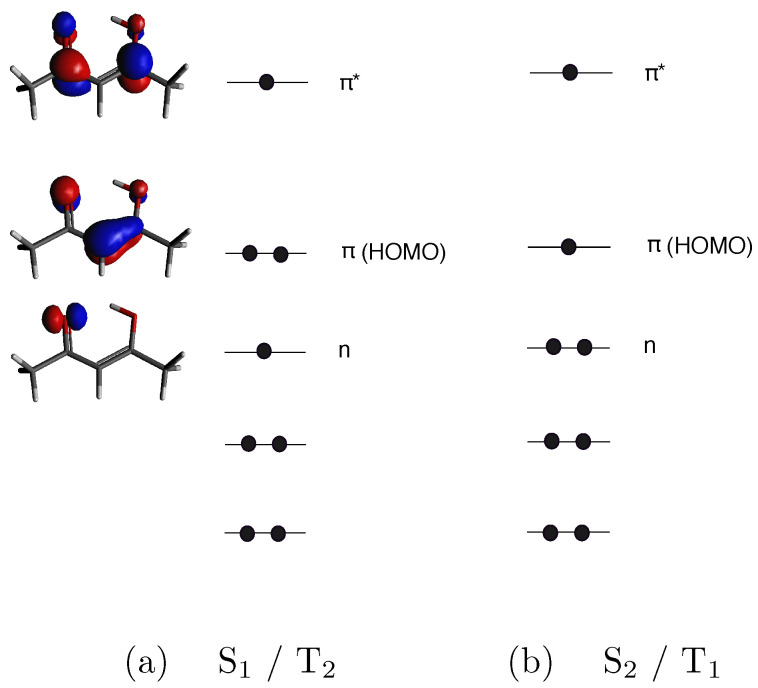
Electronic configurations of the S${}_{1}$, T${}_{2}$ (**a**) and S${}_{2}$, T${}_{1}$ (**b**) excited states of acetylacetone with the plots of the three outer valence molecular orbitals calculated with CASSCF/cc-pVDZ.

As reported in our previous work [34], four main steps involving two singlet and two triplet states can be distinguished in the acetylacetone photodynamics: (i) photoexcitation of the molecule in the enolic form (i.e., the most stable form at room temperature and in the gas phase) to the S${}_{2}$ ($\pi \pi^{*}$) bright state; (ii) conical intersection between the S${}_{2}$ ($\pi \pi^{*}$) state and the S${}_{1}$ ($n\pi^{*}$) dark state; (iii) ultrafast S${}_{1}$ ($n\pi^{*}$)/T${}_{2}$($n\pi^{*}$) crossing; (iv) internal conversion to the T${}_{1}$ ($\pi \pi^{*}$) state (see Figure 1).

The ionization of the main excited species involved in the relaxation mechanism of acetylacetone corresponds to the energy region of the valence photoelectron spectra between 3 and 8 eV. In this energy range, one can identify three different peaks, appearing, respectively, at binding energies (BEs) of 4.64, 6.04 and 7.14 eV [34]. From the ionization energies (IEs) calculated with the Multistate Complete Active-Space Second-Order Perturbation Theory (MS-CASPT2) and reported in Table 1, one can see that the lowest-lying peak can be uniquely associated with the transition from the bright S${}_{2}$ state to the first ionic state D${}_{0}$ at the S${}_{0}$ minimum geometry (S${}_{0min}$), whereas the assignment of the second peak is not so straightforward. Indeed, this feature can be associated with three different transitions, i.e., D${}_{1}\leftarrow$ S${}_{1}$, D${}_{0}\leftarrow$ T${}_{1}$ and D${}_{1}\leftarrow$ T${}_{2}$, all calculated at the S${}_{1}$ minimum geometry (S${}_{1min}$).

Such ambiguity in the assignment of the different peaks only based on the IEs values has led us to explore the possibility of distinguishing the different excited states involved in the acetylacetone photodynamics through their MFPADs.

**Figure 2:**
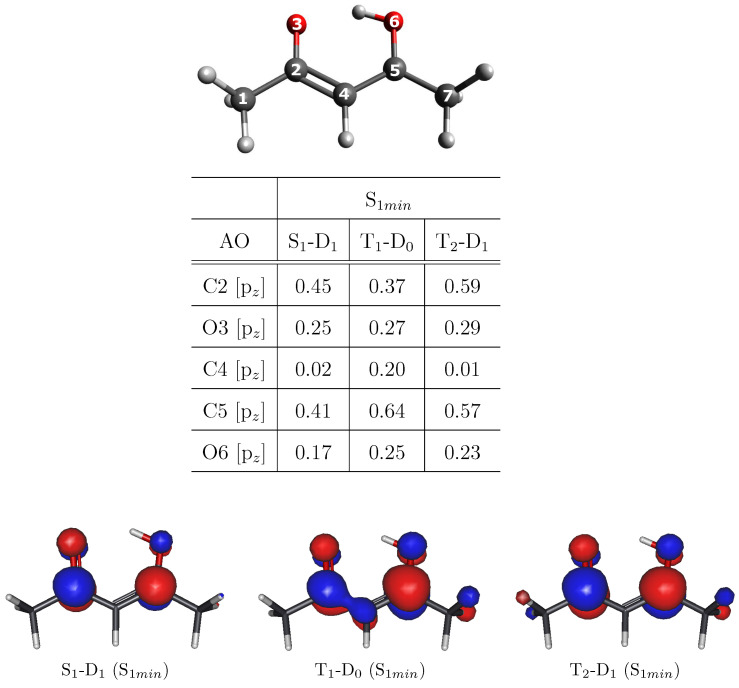
Selected coefficients of the Dyson orbitals expressed in the AO basis together with the Dyson orbital plots (CASSCF/cc-pVDZ) for the ionization from the excited states involved in the photodynamics process of acetylacetone.

In order to calculate the dipole transition moments needed to evaluate the MFPADs (see Section 2), we employed the Dyson orbital corresponding to each investigated transition. The electronic configurations of the S${}_{1}$ and T${}_{2}$ states involve an electron occupying the HOMO-1 (*n*) and an electron excited to the first virtual orbital ($\pi^{*}$), whereas the S${}_{2}$ and T${}_{1}$ states derive from the excitation of an electron occupying the HOMO (π), as sketched in Figure 1. Thus, D${}_{0}$ corresponds to the first ionic state for the configuration $\pi \pi^{*}$ (S${}_{2}$ and T${}_{1}$ states) and D${}_{1}$ corresponds to the first ionic state for the configuration $n\pi^{*}$ (S${}_{1}$ and T${}_{2}$ states). For the second peak, we calculated, at the geometry of S${}_{1min}$, the Dyson orbitals for the three transitions that can be associated with it: (i) D${}_{1}$← S${}_{1}$; (ii) D${}_{0}$← T${}_{1}$; (iii) D${}_{1}$← T${}_{2}$. Since each of the considered excited-state configurations is characterized by the presence of an electron in the $\pi^{*}$ molecular orbital, the ionized orbital would be identical for all the transitions at the single-particle level, and only modified by correlation. This affects the nature of the Dyson orbital corresponding to each transition. Specifically, the final ionic state will be the same for each couple of transitions considered and this has an impact on the possibility of clearly distinguishing excited states in such a case. As clearly shown in Figure 2, the Dyson orbital for the T${}_{1}$-D${}_{0}$ transition has a considerable contribution deriving from the C4 atom, which is negligible in the case of S${}_{1}$-D${}_{1}$ and T${}_{2}$-D${}_{1}$ transitions. For the last two, the Dyson orbitals are very similar to each other (see also AO coefficients reported in Figure 2). We will discuss below whether these differences can lead to an appreciable variation in the MFPAD profiles associated with the different excited states.

### MFPAD Profiles

**Figure 3:**
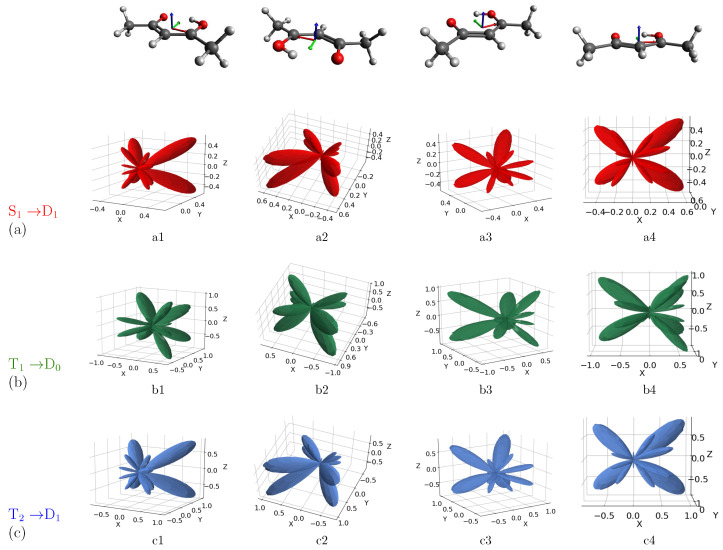
Computed MFPADs for the photoionization from the S${}_{1}$ (**a**), T${}_{1}$ (**b**), and T${}_{2}$ (**c**) excited states of acetylacetone to the corresponding first ionic state, at the selected kinetic energy of 6.04 eV. The electric field is oriented along the x axis. Orientation of the molecule is also shown on top of figure (x, y, z axes are, respectively, identified by red, green and blue colors).

We calculated MFPADs for the ionization of the excited states involved in the acetylacetone photodynamics at two selected kinetic energies of 12.09 eV and 13.19 eV, corresponding, respectively, to the binding energies of the experimental peaks, i.e., 7.14 eV and 6.04 eV (by assuming the energy of the probe fixed at 19.23 eV, as in the experimental setup). MFPADs are defined by the angles that determine both the polarization vector of the radiation and the direction of the electron momentum. Although we obtained MFPADs for all three orientations of the electric field at the two selected kinetic energies, here, we show only the most representative results, i.e., MFPADs for field orientations showing a clear possibility of discriminating the ionization processes. The remaining results are reported in the Supplementary Materials (Figures S1–S4). Figure 3 shows the MFPADs calculated for the three transitions S${}_{1}$-D${}_{1}$ (red, (a) row), T${}_{1}$-D${}_{0}$ (green, (b) row) and T${}_{2}$-D${}_{1}$ (blue, (c) row) at the selected kinetic energy of 13.19 eV (BE = 6.04 eV) and with the electric field oriented along the x-axis. The acetylacetone molecule is shown at the top of Figure 3 with the same orientation as that of the MFPADs, for ease of reading. In Figure 3, each row (a, b, c) corresponds to different views of the same MFPAD associated with the specified transition. This means that, for a selected axis orientation, the comparison of the MFPAD profiles associated with different excited states can be followed along each column.

Altogether, MFPADs for the three transitions appear to be highly anisotropic and characterized by lobes and nodes whose shape and position depend on the scattering dynamics of the photoelectrons. Starting by analyzing the case of the electric field oriented along the x-axis, and by observing the emission for the S${}_{1}$-D${}_{1}$ transition from the perspectives a2 and a4, a quatrefoil-shaped structure perpendicular to the molecular plane (xy) is clearly defined. More specifically, MFPADs are characterized by four main lobes in the half-plane y > 0, where the oxygen atoms lie. Along negative values of the y-axis, there are several additional structures of lesser extent. Therefore, the emission is more intense around the position of the oxygen atoms. By comparing the MFPADs associated with the three transitions, one can immediately notice that the profiles of S${}_{1}$-D${}_{1}$ and T${}_{2}$-D${}_{1}$ are very similar to each other but differ from the one related to T${}_{1}$-D${}_{0}$, especially in terms of intensity. Indeed, the emission profile extends to greater x and z values, while the MFPAD related to S${}_{1}$-D${}_{1}$ and T${}_{2}$-D${}_{1}$ is confined to a smaller volume.

Such difference in the MFPADs profiles is a signature of the different electronic nature of the excited states, being S${}_{1}$ and T${}_{2}$ characterized by a $n\pi^{*}$ electronic configuration, whereas T${}_{1}$ by a $\pi \pi^{*}$ configuration. In particular, going from the pair of transitions S${}_{1}$-D${}_{1}$/T${}_{2}$-D${}_{1}$ to T${}_{1}$-D${}_{0}$, the lobes on the half-plane y < 0 containing the methyl groups, as well as those in correspondence with the oxygen atoms’ position, show a change in their intensity, resulting in a profile with lobes of more comparable extension to each other. More precisely, the larger lobes shrink, while the smaller ones gain intensity, and the emission is more intense in the xz plane perpendicular to the molecular plane. Although such changes in intensity and shape are not extremely pronounced, one can in principle distinguish at least one excited state from the other two. In other words, even if we are basically considering all $\pi^{*}$ ionization, MFPADs are sensitive enough to the nature of the initial states to allow us to appreciate differences between excited states of different nature.

**Figure 4:**
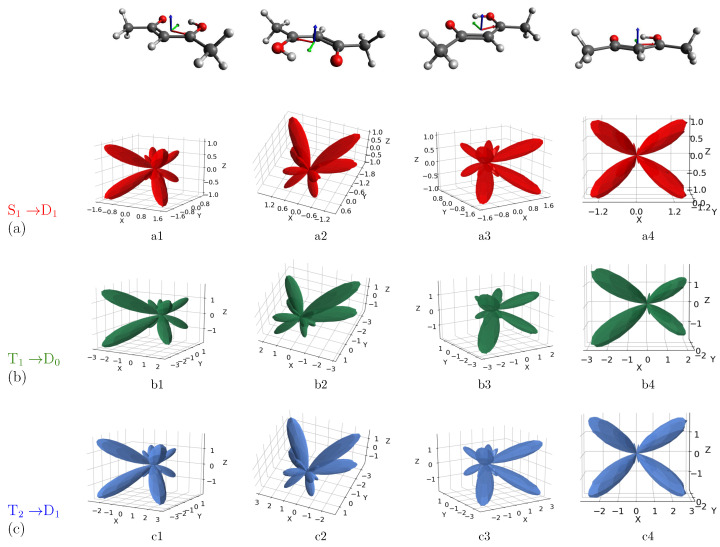
Computed MFPADs for the photoionization from the S${}_{1}$ (**a**), T${}_{1}$ (**b**), and T${}_{2}$ (**c**) excited states of acetylacetone to the corresponding first ionic state, at the selected kinetic energy of 6.04 eV. The electric field is oriented along the y-axis. Orientation of the molecule is also shown on top of figure (x, y, z axes are, respectively, identified by red, green and blue colors).

Moving to analyze the case of the electric field oriented along the y-axis (Figure 4), we observe an inversion of preferential emission with respect to the case of the electric field oriented along the x-axis, resulting in four main lobes along negative values of the y-axis, i.e., in correspondence with the position of the methyl groups. MFPADs are characterized by the presence of less intense lobes around the position of oxygen atoms, as well as by additional small lobes confined to the origin of the structure.

As already highlighted in the case of the electric field oriented along the x-axis, although the whole shape of the MFPAD is quite similar for the three transitions, some not negligible differences can be appreciated between S${}_{1}$-D${}_{1}$/T${}_{2}$-D${}_{1}$, on one hand, and T${}_{1}$-D${}_{0}$, on the other. For example, by looking at the b2 and b4 views associated with the transition T${}_{1}$-D${}_{0}$, one can appreciate a loss of symmetry along the x-axis in the emission profile. The appearance of such differences justifies the possibility of discriminating excited states by angularly resolved observables. Such possibility can be explained by considering the electronic configurations of the excited states (see Figure 1): S${}_{1}$ and T${}_{2}$ have the same electronic configuration, with HOMO-1 singly occupied, whereas HOMO-1 is doubly occupied for T${}_{1}$ state. Although all the ionizations considered are ionizations from $\pi^{*}$, differences in the initial-state electronic configuration affect the Dyson orbital and then the resulting MFPAD. A closer inspection of the Dyson orbitals (Figure 2) allows us to relate their shapes to the resulting MFPADs. More precisely, Dyson orbitals associated with S${}_{1}$ and T${}_{2}$ excited states are more symmetric in terms of the coefficients in the atomic orbitals (AO) basis. The Dyson orbital related to the T${}_{1}$-D${}_{0}$ transition, although similar with regard to the coefficients of the C5 and O6 atoms, has an additional contribution of the C4 atom. This contribution apparently “steals” part of the density from the C2-O3 side, which seems to result in smaller lobes on that side of the molecule. In our case, the differences in the final MFPAD are entirely due to the differences in the electronic configurations of the initial excited states.

By taking into account the three different orientations of the field (see SM for the results associated with the electric field oriented along the z-axis), it is clear that the possibility of discriminating between the excited states strongly depends on the orientation of the electric field. This leads to the consideration that an accurate preliminary theoretical study on MFPADs can orient the experiment by predicting the best incoming field direction for obtaining a more appreciable discrimination.

## 4. Conclusions

In pump-probe experiments studied with time-resolved photoelectron spectroscopy, the mapping of the ionization energies is often insufficient to unambiguously characterize the electronic state of the system, both due to possible closeness in energy and the fact the IEs change significantly with changing geometries. Detection of angular distributions adds more information, ideally up to the full distribution for oriented molecules. Although very demanding, recent advancements in impulsive alignment hold great promise for the immediate future. Here, we have investigated the potential of MFPAD measurements in a very difficult case, from a recent study of acetylacetone [34]. The four electronic states involved feature two electronic configurations, $n\pi^{*}$ and $\pi \pi^{*}$, due to excitation of the HOMO orbital (*n*) or the HOMO-1 (π) to the LUMO ($\pi^{*}$), both for singlet and triplet couplings. As the lowest ionizations always involve the $\pi^{*}$ orbital, in principle, all states are expected to give rise to the same photoionization cross-section, and only electron correlation may differentiate the Dyson orbital relative to the same nominal $\pi^{*}$ orbital in the different excited states. The simulations performed at the energies employed in the experiment show that while MFPADs can discriminate between the two excited configurations, the difference between $n\pi^{*}$ singlets and triplets is very small, and hardly observable (it is instead quite large for $\pi \pi^{*}$, not reported since S${}_{2}$ state is clearly identified on energetic grounds). Theoretical modeling can be useful in advance of difficult experiments to suggest the sensitivity of different observables to the process studied, and to optimize experimental conditions such as photon energy and selected angular distribution to maximize information content.

## Figures and Tables

**Table 1:** Experimental and theoretical ionization energies (IE) of acetylacetone to the first ionic state (in eV) for the investigated transitions.

Experiment [34]	4.64	6.04			7.14	
Transition	D${}_{0}$ ← S${}_{2}$	D${}_{1}$ ← S${}_{1}$	D${}_{0}$ ← T${}_{1}$	D${}_{1}$ ← T${}_{2}$	D${}_{0}$ ← T${}_{1}$	D${}_{1}$ ← S${}_{1}$
at the geometry of	S${}_{0min}$	S${}_{1min}$	S${}_{1min}$	S${}_{1min}$	T${}_{1min}$	T${}_{1min}$
MS-CASPT2[10,10] [34]	4.43	5.70	5.78	5.77	6.77	6.70

## Data Availability

Not applicable.

## Supplementary Materials

The following are available online at https://www.mdpi.com/article/10.3390/molecules27061811/s1, Figure S1: Computed MFPADs for the photoionization from the S${}_{1}$ (a), T${}_{1}$ (b), and T${}_{2}$ (c) excited states of acetylacetone to the corresponding first ionic state, at the selected kinetic energy of 6.04 eV. The electric field is oriented along the z axis. Orientation of the molecule is also shown on top of figure (x, y, z axes are respectively identified by red, green and blue colours), Figure S2: Computed MFPADs for the photoionization from the S${}_{1}$ (a) and T${}_{1}$ (b) excited states of acetylacetone to the corresponding first ionic state, at the selected kinetic energy of 7.14 eV. The electric field is oriented along the x axis. Orientation of the molecule is also shown on top of figure (x, y, z axes are respectively identified by red, green and blue colours), Figure S3: Computed MFPADs for the photoionization from the S${}_{1}$ (a) and T${}_{1}$ (b) excited states of acetylacetone to the corresponding first ionic state, at the selected kinetic energy of 7.14 eV. The electric field is oriented along the y axis. Orientation of the molecule is also shown on top of figure (x, y, z axes are respectively identified by red, green and blue colours), Figure S4: Computed MFPADs for the photoionization from the S${}_{1}$ (a) and T${}_{1}$ (b) excited states of acetylacetone to the corresponding first ionic state, at the selected kinetic energy of 7.14 eV. The electric field is oriented along the z axis. Orientation of the molecule is also shown on top of figure (x, y, z axes are respectively identified by red, green and blue colours).
