# Supplementary material for: Discrimination of Excited States of Acetylacetone through Theoretical Molecular-Frame Photoelectron Angular Distributions
Source: Molecules. 2022 Mar 10;27(6):1811. doi: 10.3390/molecules27061811 (PMC8951278; doi:10.3390/molecules27061811)
Supplement: Supplementary file 1 [file molecules-27-01811-s001.zip › molecules-1627894-supplementary.pdf]

Supplementary Materials: DISCRIMINATION OF EXCITED STATES OF ACETYLACETONE THROUGH THEORETICAL MOLECULAR-FRAME PHOTOELECTRON ANGULAR DISTRIBUTIONS

Aurora Ponzi [1*], Marin Sapunar [1,a], Nađa Došlić[1] and Piero Decleva [2*]

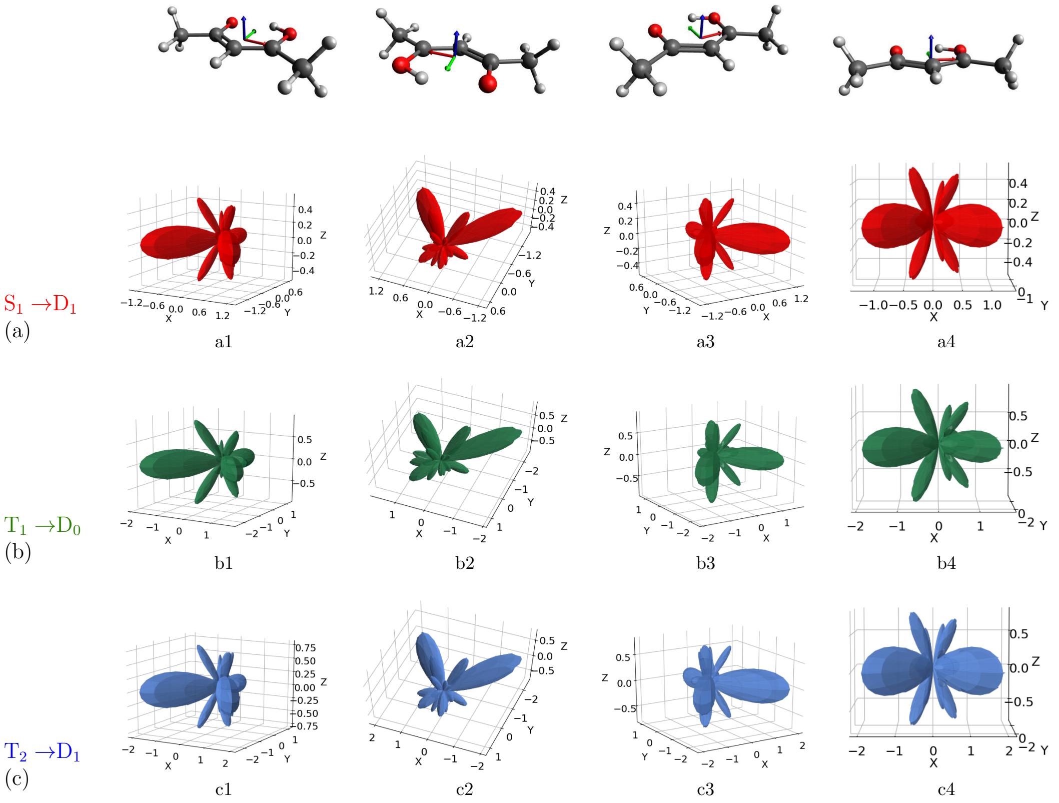

Figure S1. Computed MFPADs for the photoionization from the S_1 (a), T_1 (b), and T_2 (c) excited states of acetylacetone to the corresponding first ionic state, at the selected kinetic energy of 6.04 eV. The electric field is oriented along the z axis. Orientation of the molecule is also shown on top of figure (x, y, z axes are respectively identified by red, green and blue colours).

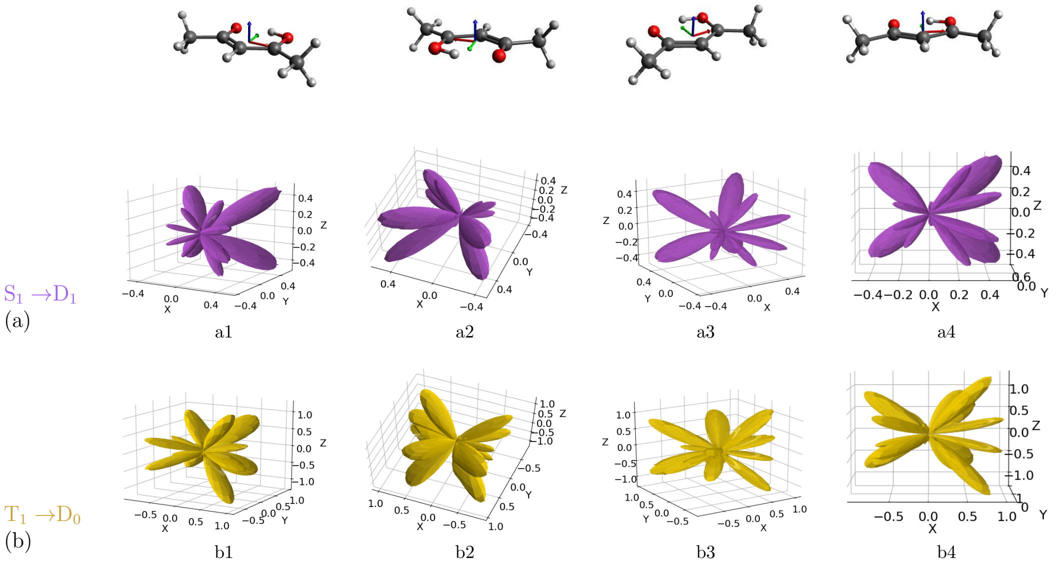

Figure S2. Computed MFPADs for the photoionization from the S_1 (a) and T_1 (b) excited states of acetylacetone to the corresponding first ionic state, at the selected kinetic energy of 7.14 eV. The electric field is oriented along the x axis. Orientation of the molecule is also shown on top of figure (x, y, z axes are respectively identified by red, green and blue colours).

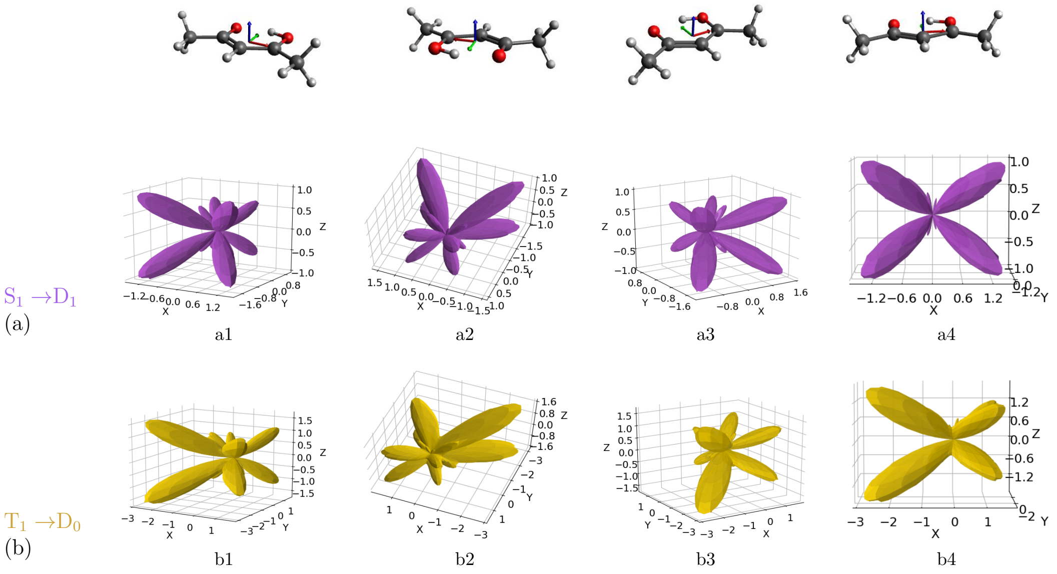

Figure S3. Computed MFPADs for the photoionization from the S_1 (a) and T_1 (b) excited states of acetylacetone to the corresponding first ionic state, at the selected kinetic energy of 7.14 eV. The electric field is oriented along the y axis. Orientation of the molecule is also shown on top of figure (x, y, z axes are respectively identified by red, green and blue colours).

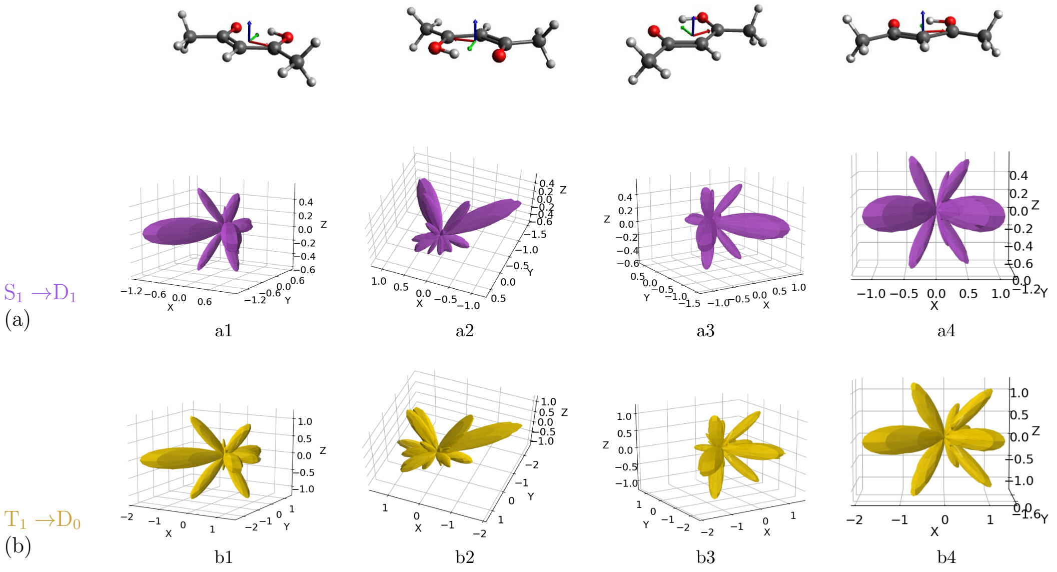

Figure S4. Computed MFPADs for the photoionization from the S_1 (a) and T_1 (b) excited states of acetylacetone to the corresponding first ionic state, at the selected kinetic energy of 7.14 eV. The electric field is oriented along the z axis. Orientation of the molecule is also shown on top of figure (x, y, z axes are respectively identified by red, green and blue colours).
